# Supplementary material for: A Critical View of the Application of the APEX Software (Aqueous Photochemistry of Environmentally-Occurring Xenobiotics) to Predict Photoreaction Kinetics in Surface Freshwaters
Source: Molecules. 2019 Dec 18;25(1):9. doi: 10.3390/molecules25010009 (PMC7017383; doi:10.3390/molecules25010009)
Supplement: Supplementary file 1 [file molecules-25-00009-s001.zip › 3.2.4_gcc-4.4.0.zip!/3.2.4_gcc-4.4.0/3.2.4_gcc-4.4.0/doc/octave/html/interpreter/Numeric-Objects.html]

Numeric Objects - Untitled


Next: Missing Data,
Up: Built-in Data Types


---

#### 3.1.1 Numeric Objects

Octave's built-in numeric objects include real, complex, and integer
scalars and matrices. All built-in floating point numeric data is
currently stored as double precision numbers. On systems that use the
IEEE floating point format, values in the range of approximately
2.2251e-308 to 1.7977e+308
can be stored, and the relative precision is approximately
2.2204e-16.
The exact values are given by the variables `realmin`,
`realmax`, and `eps`, respectively.

Matrix objects can be of any size, and can be dynamically reshaped and
resized. It is easy to extract individual rows, columns, or submatrices
using a variety of powerful indexing features. See Index Expressions.

See Numeric Data Types, for more information.
